# Observations on Functional Disorders of the Spinal Cord, and Their Connexion with Hysterical, Nervous, and Other Diseases; Illustrated by Cases, Selected Chiefly from the Reports of the Pallas-Kenry and Currah Dispensaries

**Published:** 1831-01

**Authors:** Wm. Griffin, D. Griffin

**Affiliations:** Limerick.; Limerick.


THE LONDON

### Medical and Physical Journal.

Nº 383, VOL. LXV[illegible]] JANUARY 183[illegible]. [Nº 55, *New Series.*

For many fortunate discoveries in medicine, and for the detection of numerous errors, the world is indebted to the rapid circulation of Monthly Journals; and there never existed any work to which the Faculty, in Europe and America, were under deeper obligations than to the *Medical and Physical Journal of London*, now forming a long but invaluable series.—RUSH.

### ORIGINAL PAPERS, AND CASES,

### OBTAINED FROM PUBLIC INSTITUTIONS AND OTHER AUTHENTIC SOURCES.

---

### FUNCTIONAL DISORDERS OF THE SPINAL CORD.

*Observations on Functional Disorders of the Spinal Cord, and their Connexion with Hysterical, Nervous, and other Diseases; illustrated by Cases, selected chiefly from the Reports of the Pallas-Kenry and Currah Dispensaries.* By WM. GRIFFIN, M.D. and D. GRIFFIN, Esq. M.R.C.S. Limerick.

(Continued from vol. lxv. p. 397.)

Loss of appetite is so usual a symptom in all diseases disturbing the health, that it could offer little satisfactory to the mind to state cases, in which, after all its dependence on disturbed function of the eighth, must be in some degree conjectural. This is, however, by no means true with respect to hunger, which, occurring in the course of any disease previous to convalescence, must be looked upon as symptomatic of some peculiar state of the cerebral substance at the origin of the par vagum. Thus, in cases of hydrocephalus, of apoplexy, and paralysis, where there is a partial recovery, or the disease does not become quickly fatal, the patient will frequently ask for food, and, if indulged, eat enormous quantities of the plainest diet. The following singular instance of spinal irritation, in which hunger was the first link in the chain of morbid symptoms, has occurred to us within these few days.

XXXVII. Mary Howard, aged thirty-three years, ill four weeks, complains that she becomes so hungry immediately after eating her breakfast or dinner, that she is ready to faint; her stomach then becomes sick, but she never vomits, and this is followed by pain, sometimes very severe, in the epigastrium, which usually lasts about an hour. The catamenia are regular, the bowels free, tongue a little white, and pulse natural. On examining the spine, there was considerable tenderness at the second cervical vertebra, and a slighter degree at the first and third, but none whatever in any of the dorsal; there is pain on pressure of the stomach, but none is excited there by pressure on the affected vertebræ.

Very frequently, as in this case, the sickness or nausea is accompanied by pain; sometimes almost constant, as in constant gastrodynia, sometimes occurring, like cramp, in sudden violent fits. The pain, in either instance, occurs without the nausea; but as this seemed to be the case most frequently with the former, and as the attendant cervical tenderness was almost invariably accompanied by a similar tenderness of the seventh or eighth dorsal vertebra, we were, at one period, strongly inclined to believe the gastrodynia with dorsal tenderness was superficial, a simple neuralgic affection of one of the dorsal pair, while the deep-seated cramp-like pain, with cervical tenderness, was in the stomach itself, an affection of the eighth pair, and therefore more frequently accompanied by nausea. Subsequent observation did not seem to corroborate this conjecture; nor can we yet understand why, in deranged functions of the stomach, we have sometimes tenderness of one of the cervical vertebræ only, sometimes of the seventh or eighth dorsal only, sometimes, and most frequently, of both. The fact of the dependence of the pain on irritation or disease of some portion of the spine, which is itself of great practical importance, can, at all events, be sufficiently established. We shall first quote a case from the Glasgow Journal, to show the connexion of cramp of the stomach with disease at the base of the brain.

XXXVIII. "A lady, twenty-eight years of age, was affected, about twelve hours after her first confinement, with well-marked symptoms of spasm in the stomach, and that without any apparent cause. She had taken a little arrow-root, prepared with water, and had slept for several hours, when she was suddenly awakened by excruciating pain in the region of the stomach. She struck this part with her hand, pressed upon it with all her force, and complained of great difficulty of breathing. The pulse was small and rapid, the skin cold and moist, and the countenance of a deadly paleness. Two draughts, containing full doses of laudanum, ether, and volatile tincture of valerian, were exhibited without effect; sinapisms, prepared with turpentine, were applied to the stomach and inferior extremities; and, about half an hour before death, when the powers of life were rapidly sinking, brandy was freely given. On dissection, the only morbid appearance that could be discovered by the most accurate investigation, was general softening of the cerebellum, with *vascular turgescence in the base of the brain.*"

The pain of stomach in this case was evidently symptomatic, and, as it would appear, altogether the result of organic cerebral disease, but mere irritation of the same parts, we have reason to believe, may produce similar effects. The probability of the occurrence of cramp of stomach after delivery, might in fact, be always inferred where there is great tenderness of the upper cervical, and seventh or eighth dorsal vertebra, especially in persons of hysterical habits or extreme nervous susceptibility. Tenderness in those situations, however, more commonly occasions, for some time previous to delivery, a sensation of burning at the epigastrium, generally with pain shooting from the back to the sternum, especially when the seventh or eighth dorsal vertebra is pressed on. It is often premonitory of those tormenting, and what have been called anomalous forms of hysteria, which delivery in such cases usually ushers in, and might, at the least, lead one to anticipate untoward symptoms in the confinement, and a tedious recovery after it.

The next is a case in which, like Howard's (XXXVII.), there was pain of stomach; but no tenderness of the dorsal vertebræ.

XXXIX. Joan Bryan, aged thirty-five years, was attacked, about a week since, with vomiting and pain of stomach, sometimes occurring in violent fits, like cramp, and *accompanied by troublesome cough.* There was some whiteness of tongue, and the bowels were free. On examination of the spine, tenderness of the second and third cervical vertebra was discovered. The patient recovered under the usual treatment.

The following is a well-marked case, in which, although the eighth pair was obviously the chief seat of irritation, the pain and tenderness of stomach seemed dependent on an affection of the seventh or eighth dorsal, and the cramp-like pains, or colic, (which were below the stomach,) on an affection of the lowermost dorsal nerves.

XL. Margaret Shaugnessy, aged twenty years, complained of pain at the pit of the stomach, and oppression with frequent pains in the bowels, attacking her like colic. There was a great deal of general weakness, nervousness, and loss of appetite; the pulse was quick and small, and the tongue whitish. Pressure on the lowermost dorsal vertebræ excited the colic pains at the umbilicus; on the seventh, at the pit of the stomach; on the upper dorsal, it occasioned a feeling of oppression, but not pain; on the lowest cervical it excited a suffocating sensation at the trachea, where it emerges from the chest; and higher up still, it gave a sensation of a lump and choking at the larynx, like the globus hystericus.

Many cases of hysteria, more complex than the foregoing, seem to depend altogether on alternate irritation at the trunk of the fifth and eighth pairs, or of either of these and some of the dorsal and lumbar nerves. They are frequent in our periodicals, and sometimes occasion much surmise and discussion as to their nature and origin. We shall give a few in illustration from our note-book, to show their connexion with tenderness of the spine, and one from the Journal Générale, Janvier, 1829, treated by M. Rene Prus, and entitled "a Case of Neuralgia of the Par Vagum, imitating Hysteria."

XLI. Judith Ryan, aged thirty-eight years, a nurse, after an operation performed on one of the eyelids, for the cure of entropeon, was attacked with nervous lowness and sinking. On particular inquiry, it appeared she was frequently affected in this way before. The sinkings are always preceded by a burning sensation shooting from the back to the pit of the stomach, and followed by perspiration and debility. Has frequent pain at the middle of the back, with a burning feel at the sides. Is often attacked with dysury; gets sudden, distressing headachs, with a rushing of blood and throbbing of the vessels of the neck and temples. Her mind sometimes wanders, and her recollection is imperfect; scarcely knows what is going on about her. Is often oppressed, and at times waked up at night, with a sense of suffocation at the throat and hoarse breathing, "*like a child's in the quinsy.*" Has frequent pain in the left side, but no cough. Complains much of languor and loss of appetite.

It was inferred from these symptoms that tenderness of spine would be discovered at some of the cervical vertebræ, at the seventh or eighth dorsal and at the lumbar. On examination, there was found extreme tenderness of the middle cervical. When her attention was directed to it by the pressure, she said that she always felt great pain and soreness of all the back of the neck, whenever she became affected with the croupy breathing, so that she could scarcely bear to have the muscles touched for some days. There was no tenderness on touching the middle dorsal, but it occasioned a great sense of oppression, a suffocating feel; and at the upper lumbar, pressure excited pain, both in the spot pressed on and at the affected part of the side.

The most remarkable feature in this case was the occurrence of croupy breathing as a symptom of irritation of the cervical spine, to which we have before called the reader's attention: the attack of dysury is very common where the lumbar spine is affected. We may observe, that the burning sensation at the pit of the stomach, already adverted to, was present, although there does not appear to have been tenderness at the seventh or eighth dorsal vertebra. The patient gradually recovered under the use of mild aperients, with tonics and nervines, and occasional counter-irritation at various points of the spine.

XLII. Catherine Hogan, aged nineteen, complained of pain at the right side and pit of the stomach, with cough and dyspnœa; gets fits of lowness and crying, and was often attacked with the globus hystericus and sense of sudden suffocation. The pain sometimes extended across the stomach to the left side, and was felt at the extremity of the clavicle and between the shoulders; complained of pain and numbness down the arms, generally moderate and bearable, but occasionally shooting down to the wrists with excruciating suddenness, and remaining for some hours. The left arm was more frequently affected than the right, and the pain, when acute there, was often suddenly transferred to the left jaw, exciting intense toothach. She was frequently affected with sickness of stomach and faintness, and now and then with violent fits resembling colic. These affections alternated with one another in an unaccountable and almost whimsical manner, so that the poor girl found a difficulty even in stating her complaint. "She could never tell" she said, "where her disease was, for it was more changeable than the wind." Her tongue was clean, but the pulse was quick, and she complained of thirst. The catamenia were suppressed, to which she attributed all her sufferings: her illness commenced with a fit of painful menstruation.

This patient's recovery was tedious. The case, like many of that class, might be supposed, in some degree, to have worn itself out, as it was five or six months before she attained her former health. Though medicine seemed to possess little influence in arresting or subduing the complaint, it was of material benefit in alleviating the troublesome symptoms, and restricting the mischief which they were likely to occasion. Considerable intervals of relief, and sometimes perfect intermissions, were evidently attributable to the treatment. It consisted chiefly of small, repeated blisters to the spine, with moderate bleedings, accompanied by remedies directed to the improvement of the digestive functions, and a restoration of strength and tone to the system. The blisters seldom gave more than temporary relief. Bloodletting, which (from the small, rapid, hysterical pulse, and general appearance of debility,) was not resorted to until the close of the complaint, when every thing else had failed, proved very useful, particularly, she thought, when performed on the foot; a fair evidence of the truth of Mr. Burn's observation, that complaints should be treated by the indications they present, rather than by inductions from a name, and that, where there is much affection of the respiratory organs from any cause, abstraction of blood is often advantageous.

XLIII. Catherine Keating, aged twenty-seven years, complains of pain at the middle of the sternum, with distressing oppression, fluttering at the heart, and great debility. Is seized, two or three times a week, with fits of insensibility, followed by shiverings and cold perspiration. These fits usually commence with sickness of stomach, and a sense of something rising to the throat; she then loses her sight and senses. She is always obliged to creep about with the utmost caution, as the slightest bend or motion, an inadvertent step, or a pebble coming accidentally beneath the foot in walking, brings on pain at the sternum, which shoots to the back, and thence down her limbs, and is followed by a sense of faintness. There is great tenderness of the third and seventh cervical, and of the seventh dorsal vertebræ.

The progress of this disease cannot be given, as the patient did not continue in attendance at the Dispensary; but the connexion between the hysterical affection and the state of the cord seems well marked. In these cases the attention is often directed to the neck by stiffness and pain of the muscles, as in the following.

XLIV. Bridget Keefe, aged seventeen years, was seized a week since with sickness of stomach, headach, heaviness and giddiness, so that she was in danger of falling, if she stooped. She had also cough and oppression, with some degree of feverishness. Her vision was imperfect; every thing about the room seemed misty and yellow, as if something was continually between her and the object she looked at, and she had frequent fits of lowness, with apprehension of dying. Two days since, she was attacked with pain or cramp in the back of the neck, "as if her neck was breaking," which subsided in the course of some hours; was subsequently affected with pain in the right side and pit of the stomach. As in most hysterical cases, her feelings of illness were very variable. There was tenderness of the upper cervical vertebræ, of the scalp, especially at the crown of the head, and of the eighth dorsal, at the right side. The girl recovered perfectly in a few days, under the use of purgatives, which is very common in recent cases. These are, indeed, very usually occasioned by some derangement in the digestive organs.

In the case given by M. Rene Prus (which we quote from Dr. Johnson's review,) the symptoms were so striking, that their dependence on some affection of the fifth or eighth pair could not possibly escape so well-informed an observer. It may, however, excite some surprise, that the connexion of a case so incontestibly hysterical with irritation of these nerves, should not have suggested the possibility of its existence in all similar complaints, rather than the unsatisfactory inference that neuralgia might so imitate hysteria, or hysteria neuralgia, as to occasion an utter confusion with respect to both. We venture to assert that it was like all other cases of hysteria, but a form of spinal or ganglionic irritation; and that, if an examination had been instituted, tenderness of the cervical and lower lumbar vertebræ would have been detected. This we think the strong analogy which it presents to cases already cited must sufficiently establish, but it shall be further illustrated as we proceed.

XLV. Rosina, a femme de chambre, aged thirty years, had been brought up in damp and low apartments, and had suffered severely from rheumatism at the early age of twelve years. At the age of sixteen, she was so much frightened at the approach of the enemy's troops to the capital, that she was seized with the most excruciating pains in the epigastrium, which came on in paroxysms, with intervals of ease. They were often accompanied with vomiting, and by the sensation of a solid body rising from the stomach into the throat. The paroxysms sometimes ended spontaneously by a copious flow of tears. Frequently, when the epigastric pains disappeared, the patient was tormented with pain in the dental or orbital branches of the fifth pair of nerves, but these were almost always confined to one side of the face. For these complaints, which were considered as hysterical, various remedies were employed, without any success. Such was the history given by the patient when she consulted M. Prus, fourteen years after the commencement of her sufferings. She said she did not mind the facial pains, but those in the stomach were intolerable, and rendered her life miserable. M. Prus considered the original cause as a moral one, that is, a violent mental emotion, and the phenomena of the epigastric pains, vomiting, and the globus, induced him to look on the affection as one of the recurrent branches of the eighth pair. The intermissions and the paroxysms suggested the idea of neuralgia, which was strengthened by the pain shifting from the par vagum to the fifth pair, and vice versâ. At the time of application, the pains were confined to the stomach, and that between the hours of three and half-past seven P.M. The sulphate of quinine was prescribed during the intermissions, and in five days the pains ceased.

The patient considered herself radically cured, and was in high spirits; but, on the twenty-second day afterwards, the most insufferable pain occurred in the course of the infraorbital nerve. A sinapism was applied to the arm of the same side, soon after which the pain quitted the face, and seated itself in the cubital nerve of the arm sinapismed. This was of momentary duration, and an interval of fifteen days of ease succeeded, when the facial pains recurred with great violence. A blister was now applied beneath each trochanter, with a view of driving the neuralgia to as great a distance as possible from its usual seat. The sciatic nerves of the right side became violently painful, and completely prevented the patient from walking. Three blisters, with intervals of two days between each, were applied along the course of the nerve. The pain then suddenly shifted to the left sciatic nerve, and flying blisters were again applied, with the effect of removing the pain. From this time (the middle of February, 1828,) the patient remained free from all pain, and from all the symptoms of an hysterical character before described.

The periodical returns of the pain, in the case related by M. Prus, which seemed to him particularly diagnostic of its neuralgic character, is very common in spinal disease. Even regular ague sometimes occurs as a symptom. Indeed, if there is any line of distinction to be drawn between neuralgic affections as a class, and functional disorders of the cord, it is simply that of the disease existing at the extremity or the origin of a nerve. Toothach sometimes exists as a neuralgic affection, arising from caries or disease of the tooth itself; sometimes as a functional affection of the cord, and dependent on irritation at the origin of the fifth, or some of its branches. The following interesting case presents an instance of pain, which was all through more or less periodical, and yet was never benefited by the usual remedies in neuralgic affections. At one period, it is true, when attacking the right side of the head, it was evidently agueish, preceded by a regular cold fit at a regular hour, and was readily cured by the sulphate of quinine; but this must be looked upon as a mere supervention of ague, which was very prevalent in the district at the time. The quinine was of no use whatever in alleviating the more constant headach.

XLVI. A lady, aged forty years, of a pale delicate person, a short time previous to her confinement, was affected with general œdema to an alarming degree, accompanied by headach, palpitation, tightness of chest, oppression, and a burning feel at the epigastrium. These symptoms were relieved by bloodletting, and completely removed by her delivery, which took place at the close of the year 1828. It was attended by hysterical convulsive twitchings and dangerous sinking for some hours. Before leaving her bed, she complained of violent pain shooting from the back to the sternum, whenever the infant was put to the breast. It always continued for some time, and was brought on by sitting up long. The recumbent position, and discontinuance of nursing, seemed to remove it; but there remained a tired feel, weakness in the back, want of appetite, and debility, which were after a time followed by pain under the left breast, and a recurrence of the palpitation. The frequent complaints of the back, and inability to sit up long, led to an examination of the spine; and there was found, on pressure, excessive tenderness of the seventh or eighth dorsal vertebra, especially at the left side. There appeared to be some slight prominence; to which, however, no importance was attached. Leeches and blistering were used without any material advantage.

The pain afterwards shifted to the right side, was often felt in both, and was generally more acute than the pain of back.

She sometimes described it rather as a sense of constriction, than pain; or compared it to the feeling one might experience from a walnut pressed within a tight belt. It almost always came on in the afternoon or evening, and usually continued until midnight, but with a very variable degree of acuteness.

Weeks elapsed in this way, without any obvious change; but the nervous system became gradually more irritable, and successive trains of symptoms occurred, which were not always easy of relief. She began to complain of some soreness and tightness of chest; and, on examination of the spine, great tenderness was now discovered at two other points, the second cervical and the fourth or fifth dorsal vertebra; pressure on either of which excited instant coughing. The pain of side, too, at whatever time of the day it occurred, came to be attended by severe fits of oppression, with feverishness and palpitation. Occasional headach was complained of for days, at times intense, accompanied by determination to the head, flushed face and brow, and throbbing at the temples. Having, in the early part of February, suffered from this a good deal, the cervical vertebræ were again examined, and the tenderness was so great about the second or third, that she had an uncomfortable feeling in the neck, from the slight pressure, for the whole day afterwards; and at night, when retiring to her room, felt a sudden rush of blood to her head, and dropt into the arms of a lady, on whom she was leaning, in a fit of insensibility. She lay for some time perfectly still, as in a state of syncope; but the cheeks and brow were flushed and hot, and the temporal arteries and carotids throbbing. Handkerchiefs, dipped in vinegar and water, were wrapped round the head, and in some time she awoke with a startled, agitated look, complaining of a sense of trembling (not palpitation) at the heart, and a burning heat in the head, hands, and feet. These fits of insensibility afterwards frequently followed the throbbing headach, especially after any exertion, as walking or dressing, and often alternated with acute pain of side and oppressions.

In the latter end of the spring, a new train of symptoms set in. After a slight inflammatory attack of lungs, for which some blood was taken, she became affected with a sensation as if a hair, or something of the kind, was stretched across the throat, which, after a time, grew very distressing. It was accompanied by a short troublesome cough, and eventually by a feeling of enlargement of the tongue and palate. This was suddenly followed by extreme difficulty of swallowing, with constant anxious efforts and convulsive action of the muscles of the throat; respiration became shrill, croupy, and difficult, and, when the fit reached its height, seeming imminently to threaten suffocation, she fell back in a state of quiet insensibility, out of which, after some minutes, she sprung up to repeat the paroxysm. There was a strange suddenness in the recurrence of sensibility on these occasions, an electric starting from the still lifelessness, with a wild, hurried, incoherent manner, which to her friends was excessively alarming, and was occasioned, as she afterwards informed me, by an apprehension that her complaint was hydrophobia. To that dreadful disease, indeed, in the extreme nervous excitement, the difficult deglutition, and dread of swallowing, the violent convulsive spasms in attempting it, and the affection of the respiratory system, the whole attack presented a perfect and terrific resemblance. It was relieved by antispasmodics and opiates; but weeks elapsed before the sensation in the throat completely left her.

In some time after, she became affected with periodic headach, which was very violent, but yielded in a few days to the sulphate of quinine. This was succeeded by extreme coldness of the whole person, especially of the feet and hands, and a return of headach. This was now very intense, affected chiefly the right side of the head, and was attended with flushing and throbbing of the right temple. In the evening, after the application of a few leeches, blood burst from the nose, and the hemorrhage continued for some days, occasionally diminished or interrupted by the use of astringents. The headach, though at first relieved by the loss of blood, seemed aggravated by the excessive flow; the pulse became small, hard, and jerking; the determinations more frequent, and attended by noises in the ears, ringing, and an aching hammering that was scarcely endurable. All these symptoms were removed by antispasmodics, tonics, and astringents, and an occasional blister to the neck. The bleedings from the nose were not long suppressed, however, when menorrhagia occurred, which seemed likely to renew all her distress. It yielded, though slowly and with occasional returns, to a continuance of the astringents and tonics. There was now some improvement of the general health; and, on a change of air, which was recommended, it became more obvious and decided. There seemed to be an arrest of the constant disposition to metastasis, and the frequent developement of new symptoms: and those which remained, as the pain of side and back, and the evening feverishness, were not so wearing and unendurable.

We have brought the history of this case down to the present period. The treatment, which has been partly passed over in the details of the case, that a more uninterrupted view might be given of the successive symptoms, consisted chiefly in palliating those which were most distressing, quieting the nervous irritability, and endeavouring to restore tone and strength to the general habit. The digestive organs were particularly attended to, and reclination was persevered in until within the last few weeks, when she was occasionally permitted to sit up during the day. Carriage exercise brought on headach, pain of side, and oppression; and, after a few trials, was therefore relinquished. The tender points of the spine were repeatedly leeched and blistered, with temporary relief; and eventually small caustic issues were made at each side of the vertebra, where the pain was first complained of. These were of very little use to the side, and proved so extremely painful and irritable, that they were soon allowed to heal up. In short, every plan of treatment that the complaint could possibly suggest, was tried without success. The sulphate of quinine, or carbonate of iron, never gave relief to the pain, and usually excited feverishness; and the exhibition of narcotics, if we except henbane and opium, seemed quite as unavailing. We had, indeed, ample evidence of the fact admitted by all medical writers who have met with the disease in its severer forms, that medicine has little or no direct influence over it. It requires an unusual confidence in the powers of nature, and no very limited experience, to allay our apprehensions, notwithstanding the certainty of recovery in the ordinary run of such cases, and the surprising instances which have occurred in the very worst. But in all tedious, intractable complaints, not necessarily connected with organic changes, the danger to be apprehended is chiefly from the supervention of diseases to which the system is already predisposed, and to which a new liability is created by the protracted weakness and irritation.

In concluding this case, we may remark some circumstances, which, though not given in its history, were very singular. The left side of the person was always colder than the right, and this was particularly observable when the right temple or brow was attacked with flushing and headach; a remarkable proof of the intimate relation between the developement of animal heat and the nervous influence. There was also, very generally, a great coldness of the extremities, which sometimes induced a regular rigor. There was often an instantaneous change of voice, a sudden weakness and hollowness of tone, without any obvious cause, and occasionally fits of syncope from very slight exertion. At one time, this lady, who was a very talented and refined observer, gave me an account of a change in the oppression affecting her, which was singularly illustrative of the metastasis from one portion of the respiratory system to another, so frequent in these irritative disorders. She usually, she said, felt oppressed, from a tightness or difficulty in the lungs themselves; but in this instance the lungs were free, and all her distress seemed to arise from an impossibility in expanding the walls of the chest: she could not sufficiently elevate the ribs, as in ordinary respiration. On another occasion, after a short fit of laughter, she was seized with a troublesome incessant cough, which lasted many hours, and was at last quieted by opiates. Similar attacks of cough were often brought on by any sudden affection of the mind, or by pressure on the cervical or dorsal vertebra. Subsequent to her suffering so severely from the intense headach, she described an extraordinary feeling she was for some time annoyed by: a desire that every thing should be said or done with an extravagant rapidity: when a person spoke, she thought they ought to speak infinitely faster; when they advanced towards her, their movements were disagreeably slow; and when walking herself, though unable to do so without some support, she felt hurried and desirous of precipitating herself onward. This feeling, it will be remembered, occurred also in Case XVI., and was one among many not particularised in the first case given in these papers. It was a functional affection of the sensorium, of which we have the prototype in its organic diseases. We were lately taking blood from the temporal artery of a young boy attacked by acute hydrocephalus, who, during the whole operation, had it to an extravagant degree: he kept continually crying out, "Hurry, mother! hurry, I tell you! can't you be quick?—there!—what are you doing?—hurry again! hurry! &c." It need scarce be mentioned, that in cases of insanity this is a very frequent symptom.

The foregoing history might better illustrate cases of general spinal irritation, than those in which the affection is altogether confined to the cervical portion of the cord; but returning to our subject, painful affections of stomach, it will serve to explain some symptoms which occurred in a case now about to be related. It is from M. Broussais' Journal, in which it is published as "a Case of Gastro-cerebral Inflammation, with some symptoms of Hydrophobia;" and is of sufficient interest, if it were only that it so clearly shows the importance of an intimate knowledge of complaints arising from disturbed functions of portions of the cord, and the dangerous and often fatal errors into which men of extensive practice and celebrity in their profession may be led, when unacquainted with them.

XLVII. A boy, five years of age, of vigorous but irritable constitution, was seized, on the 1st of May, with symptoms of gastro-enteritis, which were removed by leeches to the epigastrium, and diluent drinks. In the night of the 12th of May, he awoke from his sleep with a loud laugh, which was quickly followed by piercing cries, and then convulsive movements of the whole body. His face was flushed, his eyes sparkling, and he attempted to bite, and to squeeze tightly in his arms, every thing that came in his way. The parents were alarmed at hydrophobia, and summoned two physicians: when they arrived, the patient was in an interval of calm, lying without sense; his eyelids shut, but the balls rolling about underneath; pupils greatly dilated, but still sensible to light; face of a purplish cast, but still burning hot; pulse contracted, hard, and quick; carotids beating strongly. Twelve leeches were applied to the regions of the jugulars, sinapisms to the feet, cold lotions to the head. In five hours the physicians returned, and the scene was greatly altered: the little patient seemed in the jaws of death; his whole surface was pale and cold, pulse imperceptible; the respiratory movements were the only indications of life. These phenomena resulted from an excessive hemorrhage. The leechbites were stopped; stimulating frictions were applied, and some spoonsful of soup were injected into the stomach. The boy gradually recruited, and a corresponding reaction took place, when diluents were prescribed. During the succeeding day and night, he had repeated vacillations between paroxysms of excitement and alarming states of collapse. The latter, however, were the more predominant; but at the end of the third day he had become sensible: his appetite gradually reappeared: on the 22d of May he was convalescent, and the physicians took their leave.

M. Broussais regards this as a case of cerebral inflammation, and conceives the boy's life was saved by the local bleeding carried to syncope. Dr. Johnson, from whose interesting Review the case is taken, judiciously remarks that the symptoms were not those of either acute gastric or cerebral inflammation, and attributes them altogether to some powerful irritation of the gastric or intestinal nerves, affecting the nervous system, and imitating inflammatory action. The frequency of this we have already sufficiently illustrated, in cases of dental or gastric irritation, occasioning disturbance of the upper portion of the spinal cord, and so disordering either the sensorial, sensitive, or motory functions. That this child's attack originated in gastric irritation, the primary affection is a fair evidence of; and that it would have been removed at the commencement by active purgatives, with cold lotions to the head, and perhaps counter-irritation at the back of the neck, we do not entertain a doubt. The convulsive fits, attempts at biting, &c. we shall hereafter instance, in speaking of affections of the motory system particularly; the interval of calm which followed; the still appearance; the loss of sense, the flushed face and brow, the hot skin, and throbbing of the temporal and carotid arteries, were merely the usual symptoms of that insensible state into which patients fall when the functions of the brain and the vascular system are sympathetically disturbed, as in cases of hysteria, especially those of the anomalous class. Dr. Johnson comments with much truth on the little similitude which any of the symptoms bore to hydrophobia; but we have given an instance in the case preceding, of how really and perfectly even hydrophobia may be imitated, in irritation of the cervical portion of the cord. Indeed, there can be no doubt but this hysterical affection is often mistaken for its prototype.

We shall take this opportunity of offering some remarks on the doctrine of the Broussaian school, with respect to affections of the stomach, almost all of which it refers to acute or chronic inflammation, chiefly of the mucous texture. It seems to have originated from observing the frequent complication of gastric pain or tenderness with other diseases, and from the still more frequent discoveries of inflammatory appearances in post-mortem investigations. But if gastric tenderness, pain and sickness, are such common effects of spinal irritation, as to become almost necessary symptoms in disturbed states of the nervous systems; and if they may exist not only without the other usual characteristics of inflammation, but are relievable by remedies which should increase the inflammatory state, there can be little reason for the inferences deduced from their presence by the Broussaists. Morbid appearances on dissection may perhaps appear to furnish less fallacious or disputable evidence, but we cannot help thinking that, however useful and necessary pathological anatomy may be to the progress of medical knowledge, too much importance, and too much certainty, have been attached to its inductions. The study of morbid anatomy, when absolute facts alone are made the foundation of an opinion or a system, must eventually contribute to make the medical art in some sort an inductive science; but if observations are to include theories, and what are called facts are but hypothetical assumptions, we know of no more dangerous subjects upon which to venture even a conjecture. As to the morbid appearances in the stomach, for instance, every tint of colour that has ever been found has been set down to the account of inflammation. The slight crimson or florid blush, the dark red, the deep violet or brown, the purple, the slate-coloured grey, greenish grey, and the dead white, are all equally considered as its results; and yet these are not to be discovered in some cases that present all the characters of true inflammation, and are met with in others where no such disease could have existed. In Dr. YELLOWLY's excellent essay on Vascular Appearances in the Stomach,* dissections are given, in which they have been found to an intense degree in healthy criminals, executed in the prime of life, and in patients who had died with diseases of the brain, lungs, liver, or other parts, uncomplicated with any affections of stomach. Thinning of its coats, which JOHN HUNTER referred to the continuance of the digestive powers after death, and BROUSSAIS attributes to inflammation, Dr. Y. goes far to show is a natural inequality, observable in all stomachs. Even the affusion of coagulable lymph, which Dr. ARMSTRONG considers to be the most unequivocal evidence of inflammation, is with difficulty distinguishable from thickened mucus; and a coagulated portion of extravasated blood, adhering to the villous coat, may be mistaken for a coloured coagulum. All these facts would lead one to be very sceptical as to any doctrine of inflammation of stomach founded on its morbid anatomy. It is very truly observed by Dr. Yellowly, "that, in judging of the existence of external inflammation in the living body, it is not by mere redness or turgescence of vessels that the opinion is guided; but by those circumstances, in conjunction principally with pain, heat, and swelling. It does not, therefore, appear to be less necessary, for the purpose of enabling us to judge of the existence of internal inflammation, that something unequivocal in the symptoms should be superadded to the appearances submitted to our consideration, than that there should be assistance required in judging of external affections, in addition to mere colour or vascularity."

It may be said, perhaps, in favor of the Broussaian notions, that there is no reason to imagine gastritis could be so rare a disease as was formerly supposed, since the coats of the stomach must be as liable to inflammation as similar tissues elsewhere. It must, indeed, be admitted as a law in the animal constitution, that similar textures, in whatever part of

* Medico-Chirurgical Transactions, vol. iv. p. 371.

of the frame, are subject to similar affections. Thus we find the mucous membranes, wherever situated, more liable than other tissues to inflammation, terminating in purulent discharges, as the tunica conjunctiva in Egyptian ophthalmia, the urethra in gonorrhœa, the lining of the bladder in catarrhus vesicæ, and the bronchial membrane in bronchitis. The serous membranes, on the other hand, seem to be more readily attacked by inflammation of a rheumatic character, and other textures by acute, subacute, or chronic inflammation, whether of a common or specific nature. This general law, nevertheless, has its modifications and exceptions. Though similar structures be liable to similar diseases, there is the greatest possible difference as to susceptibility among them, which seems to have reference entirely to situation and function. For this reason it is that we have catarrh from cold so frequently, rather than purulent ophthalmia or gonorrhœa; that we have the serous membranes of the joints more frequently the seat of rheumatism, than those enveloping the heart or lungs. Nay, it would appear as if, in particular instances, the circumstances of situation and function altogether excluded tissues in one part of the frame from attacks to which they are obnoxious in every other. We know of no disease, as affecting the mucous membrane of the stomach, analogous to acute or chronic bronchitis. In fact, that an organ like this, whose function it is to receive and digest such an endless variety of material, and upon the proper performance of which all other functions depend, should be so constructed as to resist the ordinary sources of disorder incident to structures like itself, would appear to be a necessary provision of nature. If its lining were liable, like the mucous membrane of the eye, to be thrown into a state of turgescence and inflammation by the contact of a mote or a grain of sand, we should not have an hour of health or quiet in our lives. As light was necessary to vision, nature has taken care to adapt the tolerance of the organ to its greatest extremes; and in like manner we must suppose she bestowed on the stomach a power of deriving nutriment from an incalculable variety of food, and a capability of enduring great extremes of hunger and repletion: hence it is, indeed, that we find all the abuse it hourly sustains from the appetite of the epicure or the drunkard, so seldom wholly destroys its natural action, or excites organic disease.

If, then, gastric pain and tenderness are much oftener indicative of disturbance of the nervous functions than of inflammatory disease; if there is no certainty in the inferences drawn from the morbid anatomy of the organ; if we cannot tell, from symptoms in the living body, what appearances may be found in the dead, nor from appearances in the dead the manifestations of disease which had occurred in the living; and, finally, if we know that great susceptibility to inflammatory action in any organ, from the exercise of its ordinary functions, is contrary to the usual providence of nature, we may fairly conclude that the doctrine of gastro-enteritis is theoretic and extravagant, and that the disease is any thing but what it assumes to be, the most frequent of all the phlegmasiæ.

It would be out of place here to enter into any lengthened discussion on the general merits or demerits of the Broussaian system, which, from the great imperfection of our knowledge, the speciousness of its doctrines, and the talent and dogmatism with which they have been supported, has won disciples in every country, and within a few years spread itself over the face of the European continent. It seems to be the fate of the human mind, when searching its way through obscure, and perhaps inscrutable, subjects, to be in every age led astray by the tempting sight of some ingenious theory. The science of medicine has, in too many instances, to deplore the efforts of genius for its improvement; and we fear much it will be hereafter found in none so much as in those attempts at localizing all diseases in the coats of the stomach and bowels, and reducing all treatment to bleeding and starvation. Le Sage could have little conception, when he satirized the simplifying spirit of the profession, in the practice of Sangrado, that his caricature was to be outdone in the leeching and "*diete absolue*" of Broussais.

